# Xanthogranulomatous prostatitis: A case report

**DOI:** 10.1016/j.eucr.2025.103293

**Published:** 2025-11-23

**Authors:** Jun Yang, Yuan-Yuan Zhu, Shao-Jun Li

**Affiliations:** Department of Urology, Gansu Provincial Maternity and Child-Care Hospital, Gansu Provincial Central Hospital, Lanzhou, Gansu Province, 730050, PR China

**Keywords:** Xanthogranulomatous prostatitis, Prostatic carcinoma, Histology, Antibiotic therapy

## Abstract

Xanthogranulomatous Prostatitis (XGP) is a rare benign inflammatory condition of the prostate. Patients typically present with lower urinary tract symptoms or elevated serum prostate-specific antigen (PSA) levels. Differentiating XGP from prostate cancer (PCa) based solely on multiparametric MRI (mpMRI) findings remains challenging, as histopathological examination is the gold standard for definitive diagnosis. This case report describes a 57-year-old man diagnosed with XGP. Symptomatic remission was achieved following a 4-week course of antibiotic therapy combined with an alpha-blocker.

## Highlights

• Xanthogranulomatous Prostatitis (XGP) can perfectly mimic prostate cancer.
• Histopathological examination with CD68 immunohistochemistry is essential for a definitive diagnosis.
• We report successful treatment of XGP with a 4-week course of levofloxacin and tamsulosin.

## 1 Introduction

Xanthogranulomatous Prostatitis (XGP) is a rare benign inflammatory disease. The clinical manifestations of XGP are often highly similar to those of prostate cancer, including the detection of indurations upon digital rectal examination (DRE), symptoms of urinary obstruction, elevated serum PSA levels, and space-occupying lesions on imaging that resemble malignant tumors. The gold standard for diagnosis remains histopathological examination especially immunohistochemistry. We report a case of suspected prostate cancer with biopsy pathology suggestive of XGP that responded well to treatment with antibiotics and alpha blockers.

## 2 Case presentation

A 57-years-old man presented with 10 months history of increasing difficulty in micturition. He had hesitancy, weak flow, intermittency and increased urinary frequency. His International Prostate Symptom Score (IPSS) was 20. He had no significant past medical history, and his general physical examination was normal. He had not experienced any significant loss of weight. On digital rectal examination (DRE), left lobe of the prostate felt hard and nodular with no tenderness. His complete blood counts and renal function tests were normal. Routine urinalysis and urine culture was negative. The serum prostate-specific antigen (PSA) level was 26.91 ng/ml (normal 0–4 ng/ml). Transabdominal ultrasonography indicated that there was a 3cm cyst in his left kidney, bladder was normal and prostate was 3 × 3.5 × 5.5 cm in size and with inhomogeneous echogenicity and multiple calcification. Uroflowmetry showed mild bladder outlet obstruction with a maximal flow of 12.4 mL/sec, and there was 20ml post void residual volume on ultrasound.

**Fig. 1:**
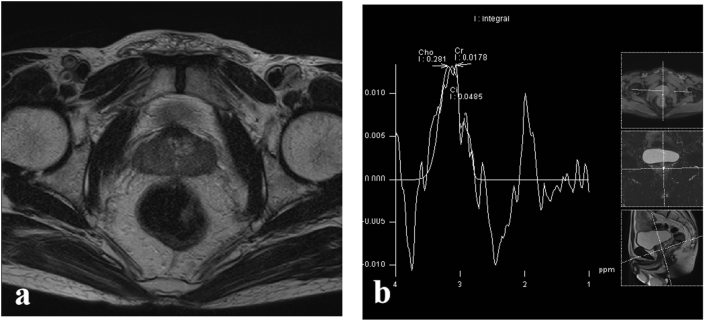
(a) T2-weighted image showing peripheral gland was of low-signal intensity. (b) MRS showing an elevated Cho peak and a reduced Ci peak in the lesion.

Multiparametric magnetic resonance imaging (mpMRI) demonstrated an enlarged and irregular prostate gland. The peripheral gland exhibited low signal intensity on T2-weighted imaging (Figure_1a). Diffusion-weighted imaging (DWI) revealed restricted diffusion in the lesion, with correspondingly low values on the apparent diffusion coefficient (ADC) map. Magnetic resonance spectroscopy (MRS) showed an elevated choline (Cho) peak and a reduced citrate (Ci) peak within the lesion (Figure_1b). This was overall reported by the radiologist as suspicious for prostate carcinoma.

**Fig. 2:**
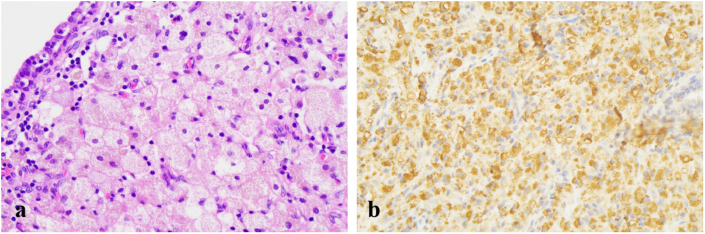
(a) Photomicrograph showing large numbers of foamy macrophages involved in the lesion (hematoxylin and eosin, × 400). (b) The inflammatory cells showing positive reaction to CD68 (immunohistochemical, × 400).

A provisional diagnosis of locally prostatic carcinoma was made. Transperineal needle biopsies were performed and with tissue fragmentation a total of 12 cores were sent. Histopathological examination of the specimen revealed numerous foamy macrophages, accompanied by infiltrates of multinucleated giant cells, leukomonocytes, and plasmocytes (Figure_2a). The immunohistochemical stains showed CD68 (+) (Figure_2b) and PSA (−). Benign prostatic glands were identified, and no evidence of malignancy was noted. It was diagnosed as XGP definitely.

Levofloxacin and tamsulosin were given for 4 weeks, the patient's symptoms improved significantly. After discontinuing the antibiotics, tamsulosin was taken intermittently. After 6 months follow-up, his IPSS was 7. His serum PSA had decreased to 1.32 ng/ml. The maximal urine flow increased to 15 mL/sec, and there was no post void residual volume on ultrasound.

## 3 Discussion

Xanrthogranulomatous inflammation is well known in the kidney and gallbladder but prostate is rare site for this lesion. XGP patients are mostly over 50 years old, but there has been a recent trend toward younger patients (e.g.,a 47-year-old case).^1^

It is currently believed that the development of XGP may be linked to chronic infections (such as recurrent urinary tract infections), obstructive processes within the prostate, immune dysregulation, and lipid metabolism disorders, leading to tissue destruction and subsequent granulomatous inflammation.^2^, ^4^ XGP may be associated with systemic granulomatous diseases (such as sarcoidosis) or allergic reactions,^3^ but the specific mechanisms remain unclear.

Multiple Gram-negative bacilli have been isolated from XGP.^5^ Chronic inflammation, prostatic calculi, or benign prostatic hyperplasia result in the narrowing or obstruction of the prostatic acini and ducts, potentially rendering bacterial cultures negative. This systematic review recommends that *for patients without abscess formation, antibiotic and alpha-blocker therapy should be commenced for up to 4–6 weeks until symptoms resolve.*^5^ Building on literature reports of successful XGP treatment with ciprofloxacin therapy,^4^^,^^6^^,^^7^ we administered levofloxacin (a related fluoroquinolone) and observed comparable clinical efficacy. Prolonged antibiotic therapy is commonly employed, largely due to the associated reduction in tissue vascularization.

The clinical manifestations of XGP are often highly similar to those of prostate cancer, including the detection of indurations upon digital rectal examination (DRE), symptoms of urinary obstruction, elevated serum PSA levels,^1^ and space-occupying lesions on imaging that resemble malignant tumors.^8^ Some cases also present with pelvic pain,^6^ recurrent hemospermia^9^ or are complicated by prostate-rectal fistulas.^3^

Multiparametric Magnetic Resonance Imaging (mpMRI) is recommended for preoperative differentiation. By analyzing the diffusion-weighted imaging (DWI) and dynamic contrast-enhanced characteristics of lesions, it can reduce unnecessary biopsies.^10^ For this case, mpMRI is limited by its low specificity. PSMA-PET, in conjunction with mpMRI, represents a valuable adjunct for distinguishing XGP from PCa. This combined imaging strategy facilitates improved delineation of suspicious prostatic lesions before treatment planning.^11^

The gold standard for diagnosis remains histopathological examination, which requires the observation of foamy histiocytes, chronic inflammatory cell infiltration, and fibrosis, while also ruling out malignant tumors.^12^ Histiocytic marker like CD68 is a useful staining technique which can differentiate prostatic carcinoma from inflammatory conditions of the prostate. Studies emphasize that even needle biopsies can be misdiagnosed as high-grade adenocarcinoma,^1^ XGP and prostatic carcinoma can also co-exist.^8^ Immunohistochemistry using prostate specific antigen may aid in resolving diagnostic dilemma.

Pharmacological Treatment: For infection-related cases, long-term antibiotic therapy can alleviate symptoms.^6^^,^^9^ We also referred to the report on the treatment of Xanthogranulomatous pyeloneohritis with antibiotics.^2^ Previous studies have indicated that Cymbalta can release pelvic pain^6^ and pollen extract can effectively control hematospermia in patients with XGP.^13^ The role of NSAIDs and corticosteroids in xanthogranulomatous prostatitis is undefined. For this case, the patient was treated with levofloxacin as antibiotic therapy. Tamsulosin was concurrently administered to alleviate voiding symptoms, resulting in a favorable therapeutic outcome.

Surgical Treatment: For refractory conditions, an operation of TURP or open prostatectomy is the primary approach. Cases complicated by abscesses can be treat with aspiration drainage. Cases complicated by prostate-rectal fistulas require a combined approach from urology and general surgery.^3^ In recent years, some scholars have recommended radical prostatectomy for localized lesions suspected of malignancy, with postoperative pathology confirming XGP,^14^ we believe that prostate biopsy and a thorough pathological diagnosis are still very necessary.

In summary, current evidence is largely based on case reports, with a lack of large-scale cohort studies, and a consensus on treatment has yet to be established. Future research needs to explore molecular markers (such as specific inflammatory factors) to improve preoperative diagnostic accuracy.^2^ In summary, the progress in XGP research remains focused on case accumulation and optimization of diagnostic techniques, emphasizing the central role of multidisciplinary collaboration and pathological confirmation, while exploring its association with systemic immune status may become a new direction. To date, the prognosis of XGP is favorable, with no reports of malignant transformation. Long-term follow-up is necessary to rule out missed diagnoses of malignant tumors.

## 4 Conclusion

XGP is a rare diagnosis, XGP has been known to mimic prostate cancer on imaging and in clinical presentation. The gold standard for diagnosis remains histopathological examination. We present a case of XGP that was initially misdiagnosed as PCa, the patient was successfully treated with 4 weeks antibiotic therapy and alpha blockers. The patient's prognosis was excellent. Nevertheless, long-term follow-up is mandatory.

## CRediT authorship contribution statement

**Jun Yang:** Writing – original draft, Investigation, Conceptualization. **Yuan-Yuan Zhu:** Investigation. **Shao-Jun Li:** Writing – review & editing, Funding acquisition.

## Ethics declarations

Written informed consent was obtained from the patient for the publication of this manuscript and accompanying images.

## Funding

This work was supported by the 10.13039/501100004775Natural Science Foundation of Gansu Province (No. 22JR11RA178).

## Conflicts of interest

None.
